# ADAPTING COMMUNITY ENGAGEMENT STUDIOS FOR AN OLDER ADULT COMMUNITY EXPERTS

**DOI:** 10.1093/geroni/igae098.1172

**Published:** 2024-12-31

**Authors:** Janelle Christensen, Shaye Kerper, Steven Albert, Maya Ragavan, Leslie Scheunemann, Mylynda Massart

**Affiliations:** University of Pittsburgh, Pittsburgh, Pennsylvania, United States; University of Pittsburgh, Pittsburgh, Pennsylvania, United States; University of Pittsburgh, Pittsburgh, Pennsylvania, United States; University of Pittsburgh, Pittsburgh, Pennsylvania, United States; University of Pittsburgh, Pittsburgh, Pennsylvania, United States; University of Pittsburgh, Pittsburgh, Pennsylvania, United States

## Abstract

The development and implementation of older adult community engagement studios (OA-CES) to promote the inclusivity of older adults in the research process and to increase the number of research studies on (and inclusive of) older adults. This project utilized University of Pittsburgh Clinical and Translational Science Institute’s (CTSI, the required CTSA Hub) success using Community Engagement Studios (CES) to connect researchers with other populations historically underrepresented in biomedical research and bring it to older adult (OA) populations. This collaboration between the Pittsburgh Pepper Center (Pitt Pepper, NIA Center Program) and CTSI created a training and orientation process for older adults to: 1. Help researchers learn about how to more effectively include older adults in the research process and overcome barriers to the inclusion of older adults in their research; 2. Select and tailor implementation strategies to counter these barriers to inclusion, ultimately creating a toolkit that can be shared with other NIA Center Programs/ CTSA Hubs. The Community Engagement Studios themselves were tailored to address needs for inclusion of older adults during recruitment and implementation of the studios: 1) sending snail mail before calling and emailing potential participants; 2) providing a two hour orientation to research ethics and expectations for Community Engagement Studios; 3) providing multiple options for transportation to and from the in-person events; 4) providing increased tech support and training for remote events (via zoom). These best practices allow for the Pepper Center and CTSI to better consult with researchers seeking to increase the inclusion of older adults.

